# Systematic review of measures and interventions for caregiver adjustment to child autism diagnosis

**DOI:** 10.1177/13623613251407305

**Published:** 2026-01-10

**Authors:** Elysha Clark-Whitney, Lucy A Tully, Adrienne I Turnell, Bridie E Leonard, Erika C Moelle, Mark R Dadds

**Affiliations:** 1 University of Sydney, School of Psychology, Australia

**Keywords:** adjustment, autism, measurement, parents, systematic review

## Abstract

Caregivers’ adjustment to their child’s autism diagnosis has important implications for child and caregiver outcomes. However, there has been substantial variability in definitions and measurement of caregiver adjustment to autism diagnosis. This study reports a systematic review of measures of caregivers’ adjustment to their child’s autism diagnosis, and the effectiveness of intervention for caregiver adjustment. A systematic review (PROSPERO CRD42023463196) was conducted according to PRISMA guidelines. Adjustment was defined as caregivers’ psychological response to their child’s autism diagnosis. Database searches yielded 6345 unique articles, which were title and abstract screened. Full text screening was completed for 428 articles. Mixed Methods Appraisal Tool (MMAT) was used to assess study quality. The review identified 78 articles, which included 42 measures of adjustment and eight interventions targeting adjustment, four of which produced significant improvement in adjustment. Study quality was mostly adequate. The review identified a need for consensus on defining and measuring caregiver adjustment. The review also identified the need for fathers, caregivers who are autistic, and caregivers of adults to be more included in adjustment research. There is preliminary evidence for interventions supporting adjustment, but further research is needed.

**Lay abstract:**

The process of understanding and accepting a child’s diagnosis of autism, known as adjustment, is important for the ongoing well-being of autistic people and their caregivers. The way that researchers have defined and measured adjustment has not been consistent. This article reports a systematic review aiming to identify how adjustment has been defined and measured in published research. The review also aimed to identify interventions that have targeted caregiver adjustment and see whether they are effective. The review identified 78 articles, which included 42 measures of adjustment and eight interventions. Four of the interventions demonstrated significant benefits for adjustment. There is a need for further research to develop a consensus regarding definition and measurement of adjustment, so that adjustment can be measured more consistently across studies. There is also a need for research looking at whether existing interventions for autism have benefits for caregiver adjustment, and to conduct more rigorous evaluations of any new adjustment interventions that are developed.

Worldwide prevalence estimates of autism range from 1% to 3% (Bradshaw et al., 2024; Nielsen et al., 2023; Wallis et al., 2023; Yang et al., 2023). While autism can be reliably diagnosed from 18 months in children with high levels of autistic traits (Benedetto et al., 2021), the global mean age of diagnosis is 5 years (van’t Hof et al., 2020). The journey from caregivers’ first concerns regarding a child’s development to receiving an official autism diagnosis takes an average of 12 to 26 months, which can delay access to services and lead to confusion and stress for caregivers (Bent et al., 2020; Rivard et al., 2021). Then, in the period immediately after their child’s diagnosis, caregivers report experiencing a complex range of emotions such as shock, grief at the loss of the future they had envisioned for their child and worry about what the child’s future will hold (Fernández-Alcántara et al., 2016; Mansell & Morris, 2004). Some caregivers also report more positive emotions such as a sense of relief at the increased understanding of the child that can come with diagnosis (Mulligan et al., 2012).

Over time, caregivers then go through a process known as adjustment to the diagnosis. Caregiver adjustment has been considered across a range of diagnoses, including cerebral palsy (Rentinck et al., 2007), Down syndrome (e.g. Clark et al., 2020; Poehlmann et al., 2005) and cancer (Long & Marsland, 2011). However, aspects of adjustment to an autism diagnosis may be distinct from adjustment to these other conditions. In particular, uncertainty regarding the cause of autism, the diagnosis of autism through clinical judgement rather than objective medical testing, and difficulty predicting long-term outcomes can make adjustment to an autism diagnosis particularly complex (M. O’Brien, 2007). There is therefore a substantial body of literature aiming to understand the process of parent adjustment to child autism diagnosis specifically. There are also a range of autism-specific measures of adjustment to diagnosis, either adapted from measures addressing adjustment to other diagnoses, or developed specifically to assess adjustment to autism diagnosis.

Across this body of research evaluating caregiver adjustment to autism diagnosis, definitions of adjustment span a range of constructs, including more practical adjustments such as managing daily hassles and changes to daily routines (e.g. Dai & Carter, 2023), as well as psychological aspects of adjustment. Even within research focusing on psychological adjustment, definitions of adjustment range from more general caregiver mental health outcomes such as stress and mood (e.g. Benson, 2012; Gau et al., 2012), to those which look more specifically at caregivers’ attitudes and emotions regarding their child’s autism diagnosis (e.g. Da Paz et al., 2018; Dolev et al., 2016). In an effort to make the current review as focused as possible, adjustment was defined here as caregivers’ psychological response to their child’s diagnosis of autism. Adjustment is discussed interchangeably in the literature as both a process and an outcome – while theoretical discussions frame adjustment as an ongoing psychological process (Negri & Castorina, 2014), measurement typically captures a snapshot of adjustment outcomes at a particular timepoint. Within the current review, the term ‘higher adjustment’ will be used to refer to more optimal adjustment outcomes (operationalised in quantitative research as higher scores on measures of adjustment) as compared to ‘lower adjustment’. However, it is important to note that adjustment does not necessarily fall on a linear scale, and that what is conceptualised as ‘higher adjustment’ may vary depending on how adjustment is defined, and indeed how autism is understood within a given study. For example, research applying a neurodiversity lens may define optimal adjustment differently as compared to research that conceptualises autism through the medical model (Kapp et al., 2013).

The variability in definitions of adjustment to autism diagnosis is likely underscored by the range of theoretical models that have been applied to understanding adjustment. Broadly, researchers have applied process models of trauma and grief, as well as cognitive models of coping. Considering the application of models of trauma and grief, it is important to acknowledge that caregivers may be adjusting not only to the day-to-day reality of parenting an autistic child but also to their assumptions about what an autism diagnosis will mean for their child and family, and to the perceived loss of the neurotypical child and the idealised future they had imagined (Gentles et al., 2020; Sinclair, 1993). For example, caregivers may assume that an autism diagnosis means that their child will never live on their own or get married, which may or may not be accurate for their child. Researchers have therefore understood adjustment as the process of working through these initial beliefs about autism and associated feelings. Some research has conceptualised the child’s diagnosis as a traumatic event (Casey et al., 2012; W. Zhang et al., 2015) and associated research has therefore utilised post-traumatic outcome measures to measure caregiver adjustment on a continuum from post-traumatic stress or post-traumatic growth. Alternatively, Kübler-Ross’ (1969) staged model of grief has been applied to conceptualise the process of adjusting as that of grieving the life parents had expected for their child. While the majority of research on caregiver adjustment to autism diagnosis has not included autistic perspectives, neurodiversity advocate Jim Sinclair emphasised that it is important for caregivers to adjust by working through their grief for the neurotypical child they expected to have, so that they can accept and value the autistic child they do have (Sinclair, 1993). In line with a grief model of adjustment, researchers have used measures which capture some, or all, of the stages of grief, such as denial, despair and acceptance (e.g. Da Paz et al., 2018; Wayment & Brookshire, 2018). However, more recent research has emphasised that adjustment is generally non-linear and does not have a finite endpoint, as caregivers continue to adjust throughout the child’s life (Negri & Castorina, 2014). In particular, caregivers’ adjustment may fluctuate as they experience intermittent reminders of their child’s difficulties, for example during major life transitions such as the child starting school or reaching adolescence; thus, caregiver adjustment to child autism diagnosis has been conceptualised as an ongoing process of *chronic sorrow* (Copley & Bodensteiner, 1987; Olshansky, 1962). Researchers have also applied the theory of *ambiguous loss*, noting that the process of adjustment is complicated by the fact that autistic individuals can have a vast range of outcomes, which means that the future that caregivers are adjusting to is more unclear as compared to a finite loss such as bereavement (Boss, 1999; Bravo-Benitez et al., 2019; M. O’Brien, 2007).

Distinct from these more process-oriented models, another body of research has focused on adjustment from the perspective of caregivers’ cognitive appraisal of their child’s autism diagnosis (Lazarus & Folkman, 1984; Rasmussen et al., 2020). Researchers have therefore measured various aspects of parental cognitions such as self-blame (Lodder et al., 2020; Mak & Kwok, 2010), perceived injustice (Wayment & Brookshire, 2018), use of coping strategies (Ayyash et al., 2023) and rumination (Wayment et al., 2019). This cognitive approach can also include understanding caregiver attitudes and beliefs. For instance, caregivers’ stigmatising beliefs about autism and towards themselves as a parent of an autistic child, have been measured as a facet of adjustment to diagnosis (Anthony et al., 2020; Chan & Lam, 2018). However, longitudinal research exploring processes of change in these cognitive aspects of adjustment has been notably limited.

Given that adjustment has been understood through many different definitions and conceptual frameworks, it is challenging to compare findings across studies of adjustment. This limits researchers’ ability to clearly identify factors that benefit or hinder adjustment, as well as adjustment’s caregiver- and child-level sequelae. Research suggests that child-level factors such as level of autism features, as well as caregiver-level factors such as cultural background and access to informal supports, may influence caregivers’ adjustment (see Naicker et al., 2023, for a review). However, findings regarding predictors of adjustment have been notably inconsistent across studies (e.g. Dolev et al., 2016; Ferenc et al., 2023; Mohammad et al., 2022; Rosenbrock et al., 2021; Wayment & Brookshire, 2018). Caregivers with greater prior knowledge of and experience with autism generally experience fewer challenges with adjustment (Gentles et al., 2020). The experience of adjustment to a child’s autism diagnosis therefore likely differs for parents who are autistic themselves, though research has yet to assess this. Adjustment appears to be important for family functioning following a child’s autism diagnosis, including caregivers’ own mental health and well-being (Da Paz et al., 2018; Dolev et al., 2016; Jones et al., 2014; Vitale et al., 2023), as well as their ability to engage sensitively with the child (Di Renzo et al., 2020; Oppenheim et al., 2007, 2024; Wachtel & Carter, 2008). However, our ability to develop a thorough understanding of adjustment and its relation to other aspects of family functioning has been hampered by inconsistencies in how adjustment has been defined and measured. Accordingly, the first aim of the current systematic review was (1a) to identify measures of caregiver adjustment to autism diagnosis, including (1b) documenting the constructs these claim to measure as well as (1c) the reported psychometric properties of the measures.

Second, while higher adjustment appears to contribute to positive caregiver mental health and caregiver–child relationship outcomes (Di Renzo et al., 2020; Dolev et al., 2016; Vitale et al., 2023), our understanding of how best to support caregivers’ adjustment is limited. There is an increasing emphasis on the involvement of caregivers in intervention for autistic individuals, especially early intervention for young children (Maglione et al., 2012). Through this caregiver involvement in intervention, the caregiver learns strategies to develop their child’s skills and/or manage the child’s challenges, such as externalising behaviour (Schreibman & Anderson, 2001; Schultz et al., 2011; Swain et al., 2025). While some studies suggest that improvements in child functioning can have flow-on benefits for caregivers, the research examining caregiver outcomes in autism intervention remains in its early stages; several reviews have failed to find a consistent effect on caregiver outcomes and have highlighted limitations of the evidence base (Conrad et al., 2021; Factor et al., 2019; Schultz et al., 2011). Augmenting caregiver training with components focused more directly on the caregiver themselves, such as training in mindfulness or acceptance-based strategies, may improve caregiver outcomes (Li et al., 2024). However, prior reviews have tended to adopt a broad focus across multiple aspects of caregiver functioning, and so are not able to offer insights into intervention effects for caregiver adjustment specifically. For researchers and clinicians to best support families along their journey of adjustment, it is important to understand what interventions are already being provided aimed at supporting caregiver adjustment, and their effects. The second aim of the current review was therefore (2a) to identify interventions for which caregiver adjustment has been evaluated as an outcome, and (2b) their effects on caregiver adjustment.

## Method

The current systematic review adhered to PRISMA guidelines (Liberati et al., 2009). The protocol for this systematic review was registered on PROSPERO (CRD42023463196).

### Search strategy

An electronic search was conducted by the first author on August 30, 2023, of the following databases: PsycINFO, Medline, Scopus, Web of Science, and the Cochrane Central Register of Controlled Trials. The keywords used were (adjust* OR accept* OR resol* OR grief OR ‘respon* to diagnosis’ OR ‘react* to diagnosis’) AND (parent* OR caregiver*) AND (autis* OR asperg* OR ASD). Subsequent searches were conducted on November 15, 2024, and October 3, 2025, to capture articles published since the initial search date. The searches were limited to peer reviewed articles published in English and to papers classified as using quantitative methods; not all databases allowed for automatic limiting by these criteria, so some articles outside of these limits were excluded manually through later screening. No limits on publication date were applied, given that the review aimed to capture how adjustment to autism diagnosis has been measured across a changing theoretical landscape. The current review did not include grey literature searching or searching of article reference lists. Articles identified through this search were uploaded into Covidence, a web-based collaboration software platform that streamlines the production of systematic and other literature reviews (Covidence Systematic Review Software, 2023). Duplicates were automatically removed by Covidence.

### Screening

Covidence software was used to screen identified articles according to the following eligibility criteria: (1) Study sample is primary or secondary caregivers of care recipient with a confirmed diagnosis of autism spectrum disorder; (2) Study includes quantitative measurement of caregivers’ psychological response to their child’s autism diagnosis; (3) Original research articles published in peer reviewed journals in English. See Supplemental Material for complete inclusion and exclusion criteria including a list of concepts included under ‘adjustment’. At each stage of screening, the first author (E.C.W.) screened 100% of articles. Four other authors (L.T., A.T., B.L., and E.M.) completed secondary screening, resulting in a total of 30% of articles double screened at each stage. Agreement for inclusion/exclusion at the title and abstract screening stage was 93%, and for full text inclusion/exclusion was 84%. For disagreements on inclusion at title and abstract screening, the article was included through to full text screening to allow for thorough evaluation. Disagreements at full text review were resolved through discussion between the two reviewers. Where the information provided in the article was insufficient to determine inclusion at full text screening (e.g. description of outcome measure was unclear), the corresponding author was contacted via email once with the request for the relevant information. Studies were subsequently excluded if no response was received within 1 month. Any duplicates that had not been automatically removed by Covidence were manually marked as duplicates and removed during screening.

### Data extraction and quality appraisal

Data extraction was completed by the first author (E.C.W.). Extracted data were organised into the following sections: Demographics, Measure information and psychometric properties (Aim 1), and Intervention design and results (Aim 2). Multiple measures of adjustment included in the same study were extracted separately where relevant.

Article quality was assessed by the first author using the Mixed Methods Appraisal Tool (MMAT; Hong et al., 2018). Articles were classified as quantitative randomised controlled trials, quantitative non-randomised studies or quantitative descriptive studies and assessed for the five criteria for the relevant study type. For each criterion, a response of ‘Yes’, ‘No’, or ‘Can’t tell’ was selected, with the percentage of ‘Yes’ responses calculated to determine the overall article quality out of 100%.

## Results

### Study selection

**Figure 1.**
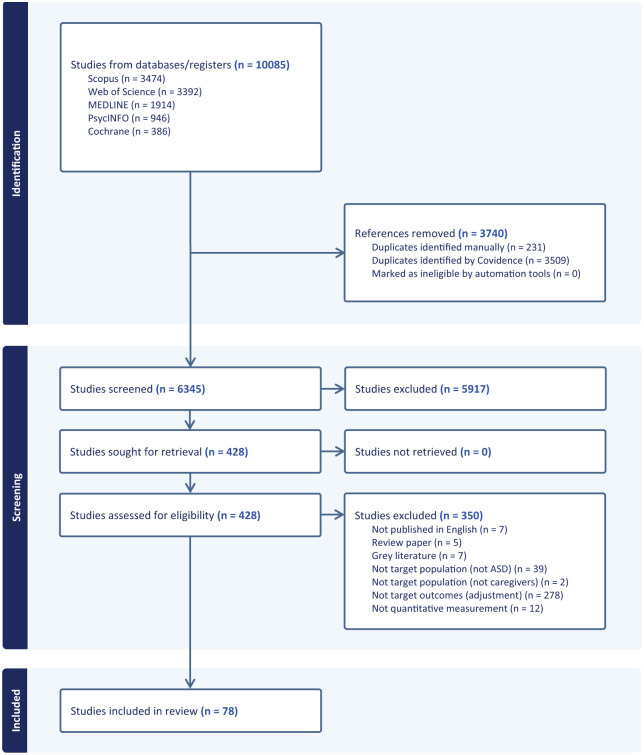
PRISMA flowchart. (Generated using Covidence).

See Figure 1 for PRISMA flowchart. A total of 3189 articles from the initial search were screened after removal of duplicates; a further 2615 and 541 were screened following the updated searches in November 2024 and October 2025, respectively. Following title and abstract screening, the full text of 428 studies was reviewed. During full text review, the authors of five articles were contacted to request for a copy of the full outcome measure to assess eligibility. One author provided a copy of the outcome measure, which was determined not to meet inclusion criteria upon review; the remaining authors did not respond and so the relevant articles were excluded due to the outcome measure as described in the article not meeting inclusion criteria. The most common reason for exclusion at full text review was that the outcome measure did not measure caregiver adjustment to the child’s autism diagnosis (*n* = 278 articles). A final sample of 78 articles was included to address Aim 1, with a subsample of eight articles with an intervention component included to address Aim 2. All articles included to address Aim 2 were also included in the sample of studies addressing Aim 1. Articles were published between 2004 and 2025. See Supplemental Table 1 for a list of included articles.

### Study quality

Study quality according to the MMAT ranged from 0% to 100%, with an average study quality of 78.03%. Study quality for each included article is reported in Supplemental Table 1. The most common reason for lower study quality was that the sample was not representative of the target population or representativeness could not be determined; this was due to articles failing to report study inclusion and exclusion criteria or providing insufficient information regarding how a diagnosis of autism spectrum disorder was confirmed in their study.

### Participant characteristics

Participant characteristics, reported in Supplemental Table 1, were excluded from analysis when not reported in an article or associated supplementary materials. Studies were conducted across 24 countries, with the highest number of studies conducted in the United States (31%). The overall sample size across 78 studies was 14,163 caregivers; however, it is important to note that articles using the same sample as another article were not excluded, and some articles utilised large autism research databases such as the Simons Simplex Collection (Fischbach & Lord, 2010). Thus, it is likely that the articles do not include 14,163 unique participants. The sample size per article ranged from 17 to 1174. Overall, 81.81% of caregivers were mothers, with 22 studies including only mothers. Seven studies included mother–father dyads of the same child. In no studies did fathers comprise the majority of the sample. Four studies reported on caregiver neurodivergence – two (Lee et al., 2024, 2025) reported whether parents were diagnosed autistic (10%–27%), self-identified autistic (5%), or questioning whether they are autistic (15%–27%), while two studies assessed parent autistic traits using a continuous measure but did not assess parent autism diagnosis (Leadbitter et al., 2025; Milshtein et al., 2010).

Children of caregivers included in the extracted articles were 79.95% male and ranged in age from 1 to 43 years. Nearly half (44%) of the included articles (*n* = 34) had a mean child age within the school age range (ages 6–12 years). No articles exclusively included adult offspring, though two studies (Lunsky et al., 2021; Montoya et al., 2024) focused on older adolescents and adults. The average child age at diagnosis across the 17 articles that reported this was 4.09 years, and the mean interval between diagnosis and completion of the measure of adjustment across the 24 articles that reported this was 40.60 months. About half of the included articles (*n* = 36, 46.15%) failed to report information about the race or ethnicity of children or parents. Twenty-nine articles included information characterising the autism traits of the sample (such as diagnosis level or mean scores on a measure of autism traits), and 41 articles included other clinical information about the children such as intellectual functioning or medical conditions.

### Aim 1 – measures of adjustment

Across the 78 studies included, 42 unique measures of adjustment were used (Aim 1a). See Supplemental Table 2 for full information regarding the included measures. All measures were questionnaires except the Reaction to Diagnosis Interview (RDI; Pianta & Marvin, 1993), which is a semi-structured interview with a coding scheme that allows for the classification of caregivers’ reaction to the child’s diagnosis as resolved or unresolved. The RDI was the most commonly used measure of adjustment, used in 13 studies. The most commonly used questionnaire measures were the Affiliate Stigma Scale (10 studies; Mak & Cheung, 2008), the Illness Perception Questionnaire Revised for ASD (7 studies; Mire et al., 2018), and the Post-Traumatic Growth Scale (7 studies; Tedeschi & Calhoun, 2004). There were 30 measures each used only in one included study. Regarding Aim (1b), the included measures spanned 22 constructs, the most common of which was perceived positive contributions of autism to the caregiver’s life. The construct assessed by each measure is listed in Supplemental Table 2. Mapping onto the broad theoretical approaches to understanding adjustment, most of these constructs focused either on the process of adjustment, or on caregivers’ cognitive appraisal of autism. Process focused measures included measures of resolution (e.g. Reaction to the Diagnosis Questionnaire; Sher-Censor et al., 2020), grief (e.g. Caregiver Grief Scale; Meichsner et al., 2016), and post-traumatic growth (e.g. Post-Traumatic Growth Scale; Tedeschi & Calhoun, 2004). Measures assessing appraisal of autism included measures of initial reaction to diagnosis (e.g. Anderberg & South, 2001), beliefs about or perceptions of autism (e.g. Attitudes Towards Autism Questionnaire; Ferenc et al., 2023) and measures of caregivers’ self-perception as a caregiver of an autistic child (e.g. Affiliate Stigma Scale; Mak & Cheung, 2008; Self-Blame & Responsibility Scale; Mak & Kwok, 2010).

Psychometric properties of included measures (Aim 1c) are included in Supplemental Table 1. The majority of measures had acceptable internal consistency (Cronbach’s α > 0.70); however, this was not reported for nine measures. Factor analysis was conducted for 11 of the measures. Only six measures were assessed for validity and six for test–retest reliability, with one of these six demonstrating non-significant correlations between Time 1 and 2 scores (Samios et al., 2009). Fifteen measures were used to evaluate change in adjustment over time, with 11 of these demonstrating significant changes over time.

### Aim 2 – interventions

Eight identified articles included an intervention targeting caregivers’ adjustment to their child’s autism diagnosis (Aim 2a). See Supplemental Table 3 for information regarding interventions and outcomes. Four intervention studies (Bravo-Benítez et al., 2024; Leadbitter et al., 2025; Lee et al., 2025; Lodder et al., 2020) used a randomised controlled trial design, while other studies used a single group design without a control group. Interventions were delivered to caregivers only (i.e. without any involvement of the child), aside from the picture book intervention (Yang et al., 2024), which briefly included children at the end of the session and required caregivers to engage children in home practice. Most interventions were delivered in group format. The majority of interventions included psychoeducation about autism and/or the adjustment process. Other intervention content included cognitive therapy strategies (Bravo-Benítez et al., 2024; Lunsky et al., 2021), Acceptance and Commitment Therapy (Leadbitter et al., 2025; Lee et al., 2025), mindfulness and acceptance-based strategies (Lodder et al., 2020; Lunsky et al., 2021) and problem-solving training (Nguyen et al., 2016). Regarding Aim (2b), the SOLACE (Lodder et al., 2020), Problem-Solving Skills Training (Nguyen et al., 2016) and caregiver grief (Bravo-Benítez et al., 2024) interventions evidenced statistically significant improvements in caregiver adjustment, which were maintained in those studies which included a follow-up timepoint. The Empower-Autism intervention (Leadbitter et al., 2025) also supported higher adjustment at 52-week follow-up as compared to treatment as usual; however, there was no measure of adjustment immediately post-intervention for this study. Medium to large effect sizes were reported for the complicated grief intervention (Bravo-Benítez et al., 2024), and a small effect size was reported for Empower-Autism (Leadbitter et al., 2025). None of the studies reported clinical significance of change.

## Discussion

This systematic review identified measures of caregivers’ adjustment to their child’s diagnosis of autism. The review’s second aim involved identifying interventions for caregiver adjustment and examining their effectiveness. The review identified 42 measures of caregiver adjustment used across 78 studies. These measures spanned 22 constructs, which spanned the main theoretical models that have been applied to understanding adjustment. The review identified eight interventions, four of which demonstrated statistically significant changes in caregiver adjustment (three compared to a control) with effect sizes ranging from small to large.

### Measurement

The included measures spanned several conceptualisations of adjustment, such as acceptance, positive contributions of the diagnosis to the caregiver’s life and post-traumatic growth. This substantial variability in how adjustment has been defined and measured was present despite this review’s specific focus on caregivers’ psychological response to their child’s autism diagnosis, excluding measures of practical adjustment or broader caregiver well-being. Amid the shifting theoretical conceptualisation of caregiver adjustment (Negri & Castorina, 2014), it is clear that the field has yet to agree on a clear definition of adjustment. This is a core limitation of the research in this area; it is not possible to validate whether a measure is accurately capturing a construct without first having a clear definition of what the construct is (Wacker, 2004). Furthermore, without clear consensus regarding what *does* and *does not* fall into a construct, researchers may fall into concept stretching, whereby related but distinct constructs blur together (Wacker, 2004). Indeed, the number of constructs assessed by the measures identified here suggests that concept stretching has likely occurred in the absence of a clear definition of adjustment to diagnosis. Inadequate definition of a construct, and concept stretching, can in turn confound researchers’ ability to identify consistent effects and draw clear conclusions, as studies that appear to measure the same construct may not be. It is therefore imperative that the next step in research investigating caregiver adjustment to child autism diagnosis is the development of a clear definition. Ideally, a consensus definition should be co-produced with autistic people and parents of autistic people, including autistic parents, and using Delphi methodology to seek expert consensus among those who work with parents of autistic children (Dalkey & Helmer, 1963).

Once adjustment is more clearly defined, further research will be needed to validate the proposed theoretical models of adjustment, and ideally to identify a model that best explains adjustment to diagnosis. While it was beyond the scope of the current study to formally identify the theoretical underpinning of each included study, a range of theoretical constructs were present, which likely contributed to variability in how adjustment was defined and measured across studies. Defining a clear theoretical model which accurately explains adjustment will support greater consistency in how adjustment is conceptualised. It was also beyond the scope of the current study to assess whether each study conceptualised autism through a medical or neurodiversity lens. This distinction will be important to explore in future adjustment research, given that the way autism itself is conceptualised in a given study will likely impact the way that adjustment to the diagnosis is understood.

The current study identified a range of measures of adjustment. In fact, nearly half the measures identified were only used in a single study, making it difficult to compare the results of those studies. It is important to note that these novel measures were often developed for studies conducted in countries that are typically underrepresented in autism research, such as Kuwait (Al-Kandari et al., 2017) and Qatar (Kheir et al., 2012). Such measures were likely developed with a goal of culturally sensitive measurement, which may indicate a need for cross-cultural validation of more commonly used measures of adjustment.

Many of the measured constructs appear to assess only part of the construct of adjustment. For example, grief measures may focus only on negative emotional reactions to diagnosis, whereas benefit-finding measures may focus only on caregivers’ positive emotions. While it is challenging to make specific recommendations before first clearly defining adjustment, it seems important that measures of adjustment should aim to capture both positive and negative reactions to diagnosis. It is not sufficient to assume that the absence of a positive reaction constitutes a negative reaction, or vice versa, given that caregivers commonly report experiencing mixed emotions in response to their child’s autism diagnosis (Anderberg & South, 2021; Makino et al., 2021). Where studies have captured multiple facets of adjustment through the use of subscales, these have shown differential relations to other outcomes (e.g. Da Paz et al., 2018; Lee et al., 2025). This suggests a need for further research to confirm whether these subsumed concepts are indeed components of adjustment. If so, adjustment may be best understood as a multidimensional construct that includes both positive and negative feelings towards a child’s autism diagnosis, and the process by which these feelings change over time.

Overall, the available information regarding the psychometric properties of adjustment measures was lacking. Very few studies reported psychometric properties beyond internal consistency. It is therefore unclear whether existing measures of adjustment have adequate test–retest reliability or can be used to identify changes in adjustment over time. This limits the utility of these measures for longitudinal or intervention research, which is particularly problematic given that adjustment is conceptualised as a dynamic construct. In addition, very few studies examined measures’ construct validity, either by comparing them to existing adjustment measures or using divergent validity to confirm that the measure is not tapping into a construct other than adjustment. This limited validation of adjustment measures likely follows from the lack of a clear definition, as discussed above. Following agreement on a definition of adjustment, further research evaluating the measurement properties of relevant measures would then allow for the recommendation of gold-standard measurement tools through rigorous assessment using a system such as PRISMA-COSMIN (Elsman et al., 2024).

While a clear conceptual definition of adjustment is clearly needed, the current review nevertheless offers some insight into currently available measures of adjustment. The most commonly used measure of adjustment was the Reaction to Diagnosis Interview (RDI; Pianta & Marvin, 1993). It is important to note that this measure was originally developed and validated with parents reacting to their child’s diagnosis of cerebral palsy. Despite its extensive use in autism research, the RDI is yet to be formally validated in caregivers of autistic children, though some studies in this population have demonstrated associations of RDI resolution status with conceptually related variables such as quality of caregivers’ interactions with the child (e.g. Dolev et al., 2016; Wachtel & Carter, 2008). The RDI has been shown to be suitable for use soon after diagnosis, as well as months to years after, and has demonstrated sensitivity to change over time with caregivers of autistic children (Poslawsky et al., 2014). It is, however, notable that the RDI asks about caregivers’ reaction to the child’s ‘diagnosed condition’ without focusing specifically on the diagnosis of autism; therefore caregivers’ responses may confound adjustment to autism diagnosis with adjustment to co-occurring conditions such as intellectual disability or Attention-Deficit Hyperactivity Disorder. It is also important to note that use of the RDI requires training of interviewers and coders. There is also an additional time burden for caregivers associated with completing an interview as compared to a questionnaire. As such, the RDI may not be suitable for use in larger-scale studies. The Reaction to Diagnosis Questionnaire (Sher-Censor et al., 2020) was developed based on the RDI to address the limitations of an interview format. This measure was used in four studies identified through this review, and demonstrated sensitivity to change through the Empower-Autism intervention (Leadbitter et al., 2025), however has yet to be formally validated with caregivers of autistic people.

Of the three most commonly used questionnaire measures, the Affiliate Stigma Questionnaire (Mak & Cheung, 2008) demonstrated adequate test–retest reliability, as well as sensitivity to intervention impact (Lodder et al., 2020). The Illness Perception Questionnaire Revised for ASD (Mire et al., 2018) has not been used to assess change over time, and demonstrates adequate test–retest reliability for most but not all subscales (Norozi et al., 2023). Test–retest reliability has not been reported for the Post-Traumatic Growth Scale (Tedeschi & Calhoun, 2004). Furthermore, intervention effects as assessed by this measure were non-significant (Bravo-Benítez et al., 2024) despite significant intervention benefits as measured by the Caregiver Grief Scale (Meischner et al., 2016), calling into question whether the Post-Traumatic Growth Scale is sensitive to intervention effects in this population. Therefore, the Affiliate Stigma Questionnaire may tentatively be recommended for use in future studies. However, it is important to note that it only captures one aspect of adjustment: the extent to which caregivers feel stigmatised as a result of their child’s autism diagnosis. Other studies have suggested that adjustment may have multiple facets, each with unique relations to other aspects of caregiver and child functioning. For example, the Adjustment to the Diagnosis of Autism Questionnaire (ADAQ; Da Paz et al., 2018) contains three subscales – despair, self-blame and acceptance – which were identified using factor analysis. These subscales demonstrated different concurrent and longitudinal associations with other aspects of caregiver mental health and well-being (Da Paz et al., 2018). Use of a more nuanced measure such as the ADAQ may therefore be recommended for studies aiming to measure adjustment. However, it is important to note that the ADAQ has, to date, only been used in two studies and has only been formally validated with mothers. Further research demonstrating replicable psychometric properties of the ADAQ, especially in fathers, would therefore be warranted before this, or another measure, can be recommended for widespread use in research and clinical practice.

While the quality of the included studies was generally acceptable, there are notable gaps in the identified research. First, many studies failed to report on the criteria used to confirm the child’s autism diagnosis. This limits the conclusions that can be drawn about the similarity of samples across studies. The current review also identified that research into caregiver adjustment is lacking for caregivers of autistic adults. In caregivers of adults who were diagnosed with autism as children, investigating adjustment many years after diagnosis could enrich our understanding of the long-term trajectories of adjustment. This is particularly important to understand in the context of the significant variability in outcomes for adults with autism (Simonoff et al., 2020). It is also important to recognise that the lack of research into adjustment among caregivers of autistic adults is positioned within a context in which research about autism in adults is more limited overall as compared to that in children. In fact, research focusing on adults has been identified by stakeholders as a priority for ongoing autism research (Frazier et al., 2018). There is also increasing interest in understanding autistic adults’ own adjustment to their diagnosis (de Broize et al., 2022); however, this was outside the scope of the current review. Only two of the included studies assessed whether caregivers themselves were autistic. Given that autism is a highly heritable condition, many caregivers of autistic children are autistic themselves (Tick et al., 2016). Research is yet to examine how parents who are diagnosed autistic, or who may be questioning whether they are autistic but not yet diagnosed, adjust to their child’s autism diagnosis, which represents a notable gap in this body of research.

Fathers were also notably underrepresented across articles identified in the current review. In fact, over a quarter of the included articles focused exclusively on mothers. This same lack of father inclusion is evident across research with caregivers of autistic children (Brown et al., 2021) and parenting research more generally (Parent et al., 2017). Fathers may experience challenges with distinct aspects of caring for an autistic child (Ornstein Davis & Carter, 2008; Vernhet et al., 2022). It would therefore be misguided to assume that research focusing predominantly on mothers’ adjustment to their child’s autism diagnosis can be applied to fathers. This necessitates validation of adjustment measures specifically with fathers and a focus on prioritising father inclusion in subsequent research.

### Intervention

The current review identified a small number of interventions targeting caregiver adjustment to their child’s autism diagnosis, which demonstrated mixed effectiveness. There is insufficient evidence on the basis of the included interventions to recommend any specific intervention modality. Psychoeducation was the most commonly-included element across interventions, however not all interventions demonstrated significant benefits of psychoeducation. Some, but not all, of the cognitive therapy and mindfulness-acceptance based programmes showed benefits. This finding aligns with a recent review showing promise for mindfulness-acceptance based programmes to support other mental health outcomes in caregivers of autistic people (Li et al., 2024). The problem-solving skills training intervention (Nguyen et al., 2016) also demonstrated significant change in adjustment; however, there was no control group for comparison. It is also important to note that the problem-solving skills training was the only individually administered intervention, and so it is not clear whether providing caregivers with one-on-one clinician support may have facilitated improvements regardless of the programme content (Baier et al., 2020). Overall, it is promising that the small body of interventions included here suggest that caregiver adjustment challenges may be amenable to change through a relatively brief targeted intervention. Further research is needed to better understand which intervention modalities or components reliably support adjustment, and to more rigorously evaluate intervention effects.

Importantly, though, evaluating the effectiveness of any intervention requires the use of an outcome measure that is appropriately validated with this population, reliable, and sensitive to change over time. Given the limitations of current measurement identified through Aim 1, it is clear that validation of a gold-standard measure of caregiver adjustment to autism diagnosis is required before interventions can be appropriately evaluated.

It is important to note that almost all of the included intervention studies specifically targeted caregiver adjustment or other aspects of caregiver mental health. The exception to this is the AutInsight intervention (Lee et al., 2025), which aimed to improve caregivers’ understanding of their autistic child and the parent-child relationship. However, AutInsight did not support significant improvements in adjustment, despite significant benefits for child conduct problems and prosocial behaviour. There were no studies identified which evaluated changes to caregiver adjustment through caregiver-mediated intervention focused on child behaviour or skills. Indeed, the potential for caregiver-mediated intervention to support adjustment should be viewed cautiously given limited evidence of benefits for other aspects of caregiver mental health (Conrad et al., 2021; Factor et al., 2019; Schultz et al., 2011). Nevertheless, it may be premature to develop targeted adjustment interventions without first exploring whether there are benefits to adjustment from the child-focused interventions that many caregivers of autistic individuals already engage in. In such interventions, caregivers are provided with psychoeducation about autism and are trained in skills that can help them manage day-to-day challenges such as their child’s tantrums and non-compliance, and to build their child’s skills (Schreibman et al., 2015). It is therefore possible that engaging in such interventions may facilitate caregivers’ adjustment by way of increasing caregivers’ knowledge and skills in relating effectively to their child, and reducing daily stressors (Dawson-Squibb et al., 2019). In line with this, caregivers engaged in qualitative research have described taking practical steps to support their child’s development, such as engaging in intervention, to have benefits for their adjustment to their child’s diagnosis (Downes et al., 2022; Rabba et al., 2019). By exploring how caregiver-mediated interventions might already be supporting caregiver adjustment, researchers can better understand whether there is a remaining need to augment such programmes with targeted content or to develop standalone interventions for caregiver adjustment.

In line with this, it is important to recognise that the majority of caregivers do successfully adjust to their child’s autism diagnosis (Gentles et al., 2020; Poslawsky et al., 2014). This points to the potential appropriateness of a stepped-care approach whereby basic support for caregiver adjustment could be integrated within existing diagnostic and early support services, with caregivers showing greater adjustment challenges then stepped up to increasingly more intensive supports such as the interventions identified in this review. A stepped-care plan for adjustment support could be easily embedded within the broader stepped-care approach to autism support recommended by the Lancet Commission (Lord et al., 2022). It may also be important to identify predictors of adjustment challenges, to understand whether preventive support for adjustment can improve families’ outcomes. Critically, applying a stepped approach to supporting adjustment requires use of a valid and reliable measure of adjustment to identify when caregivers need to be stepped up or down the levels of support, again highlighting the identification of a gold standard measure of caregiver adjustment as the necessary next step.

## Conclusion

The current review has identified a series of next steps that are needed to improve research on caregiver adjustment to autism diagnosis. It is imperative that researchers first agree on a clear definition of adjustment, including what is and is not included in this construct. Without this first step, the development and validation of relevant measures is misguided, given that it is not yet clear what is being measured. The field may then benefit from validation of theoretical models of adjustment. Consensus on key measures of adjustment is also needed to increase consistency in measurement across studies. These measures would require validation, including in underrepresented groups such as fathers, autistic parents and caregivers of autistic adults, and in a range of cultures. There is also a need to examine whether existing supports, such as caregiver-mediated interventions, are efficacious in improving adjustment. If existing interventions do not evidence sufficient benefit for caregiver adjustment, the small group of intervention studies identified in the current review reveal potential avenues for the development of more targeted support. Learnings from these identified next steps would allow optimal support for caregiver adjustment to be integrated within personalised care pathways following autism diagnosis (Lord et al., 2022), moving towards the broader goal of improving the well-being of autistic people and their families.

## Supplemental Material

sj-csv-1-aut-10.1177_13623613251407305 – Supplemental material for Systematic review of measures and interventions for caregiver adjustment to child autism diagnosis Supplemental material, sj-csv-1-aut-10.1177_13623613251407305 for Systematic review of measures and interventions for caregiver adjustment to child autism diagnosis by Elysha Clark-Whitney, Lucy A Tully, Adrienne I Turnell, Bridie E Leonard, Erika C Moelle and Mark R Dadds in Autism

sj-docx-2-aut-10.1177_13623613251407305 – Supplemental material for Systematic review of measures and interventions for caregiver adjustment to child autism diagnosis Supplemental material, sj-docx-2-aut-10.1177_13623613251407305 for Systematic review of measures and interventions for caregiver adjustment to child autism diagnosis by Elysha Clark-Whitney, Lucy A Tully, Adrienne I Turnell, Bridie E Leonard, Erika C Moelle and Mark R Dadds in Autism

sj-pdf-3-aut-10.1177_13623613251407305 – Supplemental material for Systematic review of measures and interventions for caregiver adjustment to child autism diagnosis Supplemental material, sj-pdf-3-aut-10.1177_13623613251407305 for Systematic review of measures and interventions for caregiver adjustment to child autism diagnosis by Elysha Clark-Whitney, Lucy A Tully, Adrienne I Turnell, Bridie E Leonard, Erika C Moelle and Mark R Dadds in Autism

sj-pdf-4-aut-10.1177_13623613251407305 – Supplemental material for Systematic review of measures and interventions for caregiver adjustment to child autism diagnosis Supplemental material, sj-pdf-4-aut-10.1177_13623613251407305 for Systematic review of measures and interventions for caregiver adjustment to child autism diagnosis by Elysha Clark-Whitney, Lucy A Tully, Adrienne I Turnell, Bridie E Leonard, Erika C Moelle and Mark R Dadds in Autism

sj-xlsx-5-aut-10.1177_13623613251407305 – Supplemental material for Systematic review of measures and interventions for caregiver adjustment to child autism diagnosis Supplemental material, sj-xlsx-5-aut-10.1177_13623613251407305 for Systematic review of measures and interventions for caregiver adjustment to child autism diagnosis by Elysha Clark-Whitney, Lucy A Tully, Adrienne I Turnell, Bridie E Leonard, Erika C Moelle and Mark R Dadds in Autism
