# Supplementary material for: Systematic review of measures and interventions for caregiver adjustment to child autism diagnosis
Source: Autism. 2026 Jan 10;30(4):866–83. doi: 10.1177/13623613251407305 (PMC13005899; doi:10.1177/13623613251407305)

Supplement: Inclusion and exclusion criteria

Inclusion criteria

1) Study sample is primary or secondary caregivers of a child or adolescent/adult offspring with a confirmed diagnosis of autism spectrum disorder according to the Diagnostic and Statistical Manual of Mental Disorders, Fifth Edition (DSM-5; American Psychiatric Association, 2013), or diagnosis of pervasive developmental disorder-not otherwise specified or asperger's syndrome if the study was conducted prior to the publication of the DSM-5 in 2013. Includes permanent full-time primary or secondary caregivers (e.g., biological parents, adoptive parents, step-parents, other relatives acting in the primary or secondary caregiver role); 2) Study includes quantitative measurement of caregivers' psychological response to their child’s autism diagnosis. See Supplementary Figure 1 below for a full list of included and excluded outcomes; 3) Original research articles published in peer reviewed journals in English.

Exclusion criteria

1) Not published in English; 2) Grey literature (i.e., studies from non-peer reviewed sources, conference proceedings, dissertation papers, study protocols or treatment manuals); 3) Not target population as per above inclusion criteria. Studies including caregivers of children with “features of autism”, “suspected autism” or “increased likelihood of autism” were not considered suitable for inclusion in the current study, given the focus on studying adjustment to confirmed autism diagnosis; 4) Not targeting key study outcomes. Studies were excluded if they only included measurement of the following constructs (without measurement of psychological response to autism diagnosis): other aspects of the caregiving experience (e.g., stress, burden); measures of caregiver response to features associated with autism (e.g., challenging behaviour) rather than the concept of autism itself; measures of practical aspects of adjustment (e.g., changes to daily routine) that did not involve thought, emotions or beliefs; measures of other aspects of adjustment that did not pertain to perception of autism diagnosis (e.g., broader family adjustment measures). See Supplementary Figure 1 below for a full list of included and excluded outcomes; 5) Qualitative research without quantitative measurement.

Supplementary Figure 1: Included and excluded outcome variables


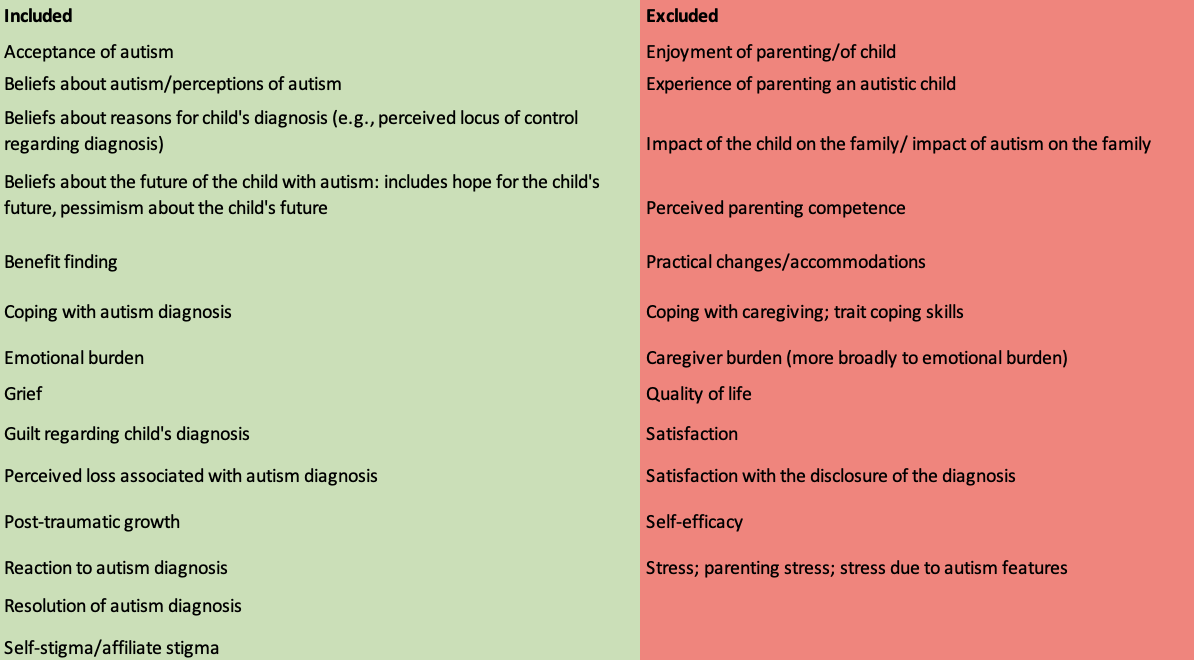

Supplement: sj-docx-2-aut-10.1177_13623613251407305 – Supplemental material for Systematic review of measures and interventions for caregiver adjustment to child autism diagnosis [file sj-docx-2-aut-10.1177_13623613251407305.docx]
