# Supplementary material for: Systematic review of measures and interventions for caregiver adjustment to child autism diagnosis
Source: Autism. 2026 Jan 10;30(4):866–83. doi: 10.1177/13623613251407305 (PMC13005899; doi:10.1177/13623613251407305)
Supplement: sj-pdf-3-aut-10.1177_13623613251407305 – Supplemental material for Systematic review of measures and interventions for caregiver adjustment to child autism diagnosis [file sj-pdf-3-aut-10.1177_13623613251407305.pdf]

Supplement Table 2. Measures of caregiver adjustment to their child's autism diagnosis.

**Measure name**	**Acceptance of Children with Autism**	***Adjustment to the Diagnosis of Autism Scale***	**Affiliate Stigma Scale**	**Appraisal of Life Events Scale**	***Attitudes Towards Autism Questionnaire***
Original citation	Harnum et al., 2007	*Da Paz et al., 2018*	Mak & Cheung, 2008	Cappé et al., 2017	*Ferenc et al., 2023*
Adapted from	N/A	N/A	N/A	Ferguson et al., 1999	N/A
Construct measured	Acceptance of autism	Adjustment	Self-stigma	Appraisal of stress from raising an autistic child	Perceptions of autism
Number of studies used	1 (Anthony et al., 2020)	2 (Abd Latif et al., 2023; Da Paz et al., 2018)	10 (e.g., Anthony et al., 2020; Cheung et al., 2019)	1 (Downes et al., 2022)	1 (Ferenc et al., 2023)
Languages available	English	English, Malay (Abd Latif et al., 2023)	Chinese, English, Arabic (Salami & Alhalal, 2024)	French	Polish
Type of measure	Questionnaire	Questionnaire	Questionnaire	Questionnaire	Questionnaire
Subscales	None	Despair, self-blame, acceptance	Affective, cognitive, behavioural<br>Internalized stigma; interactive stigma (Ting et al., 2018 version)	Threat, challenge, loss	None
**Psychometric properties***					
Number of items	7	29	22<br>Ting et al., 2018: 12<br>Lodder et al., 2020: 19	16	10
Cronbach's alpha	0.61	0.74-0.80<br>Subscales: 0.40-0.79	0.68-0.95	Subscales: 0.77-0.79	0.93
Test-retest reliability	Not reported	Not reported	Yes	Not reported	$r = 0.84$
Demonstrated sensitivity to change?	Yes	Yes	Yes	Yes	Not reported
Demonstrated validity?	Not reported	Not reported	Not reported	Not reported	Yes
Conducted factor analysis?	Not reported	Yes	Yes	Not reported	Not reported

Measure name and original citation italicized where the measure was first reported in a study included in the current review. 'Original citation' indicates that first use of the measure in the form used to measure adjustment in included studies; where this was an adaptation of a previous measure (e.g., a measure was reworded to ask about autism), the previous measure is cited as 'Adapted from'.
*Psychometric properties as reported in studies included in the current review; does not report original psychometric properties of measure with populations other than caregivers of autistic offspring. Ranges are reported where the measure was used across multiple included studies.

**Measure name**	***Autism Acceptance Scale***	***Benefit Finding***	***Benefit Finding Scale***	***Benefit Finding Scale for Parents of a Child with Asperger's Syndrome***	**Burden Assessment Scale – Mental Burden Subscale**
Original citation	*White, 2009*	*Pakenham et al., 2004*	*Ekas et al., 2015*	*Samios et al., 2009*	Reinhardt & Horowitz, 1984
Adapted from	Zuk et al., 1961	N/A	Carver & Antoni, 2004	N/A	N/A
Construct measured	Acceptance of autism	Positive contribution of autism to life	Positive contribution of autism to life	Positive contribution of autism to life	Mental burden of autism
Number of studies used	1 (White, 2009)	1 (Pakenham et al., 2004)	4 (e.g., Lovell & Wetherell, 2020; Vitale et al., 2022)	2 (Samios et al., 2012)	1 (Mohammad et al., 2022)
Languages available	English	English	English	English	English
Type of measure	Questionnaire	Questionnaire	Questionnaire	Questionnaire	Questionnaire
Subscales	None	None	None	Global benefit finding; new possibilities, personal growth, appreciation, spiritual growth, positive effects of the child, greater awareness.	Only Mental Burden subscale used
**Psychometric properties***					
Number of items	7	3	17	40	10
Cronbach's alpha	Not reported	Not reported	0.87-0.92	0.95<br>Subscales: 0.71-0.93	Not reported
Test-retest reliability	Not reported	Not reported	Not reported	Not significant	Not reported
Demonstrated sensitivity to change?	Not reported	No	Not reported	Not reported	Not reported
Demonstrated validity?	Not reported	Not reported	Not reported	Yes	Not reported
Conducted factor analysis?	Not reported	Not reported	Not reported	Yes	Not reported

Measure name	Caregiver Grief Scale	Coping Orientation to Problems Experienced Inventory (COPE/Brief COPE)	Coping Health Inventory for Parents: Coping Pattern III (understanding the health situation)	Health Specific Locus of Control measure – Parental Control Subscale	Illness Cognition Questionnaire
Original citation	Meichsner et al., 2016	Carver, 1997; Carver et al., 1989	McCubbin et al., 1981	Lau & Ware, 1981	Sint Nicolaas et al., 2016
Adapted from	N/A	N/A	N/A	N/A	N/A
Languages available	English, Spanish (Bravo-Benítez et al., 2021)	English, Spanish (Moran et al., 2010)	English	English	English
Construct measured	Grief about autism	Strategies used to cope with diagnosis	Perceptions about autism	Locus of control regarding autism	Perceptions about autism
Number of studies used	1 (Bravo-Benítez et al., 2024)	3 (e.g., Montoya et al., 2024; Roselló et al., 2022)	1 (Hall & Graff, 2011)	1 (Wayment & Brookshire, 2018)	1 (Chen et al., 2025)
Type of measure	Questionnaire	Questionnaire	Questionnaire	Questionnaire	Questionnaire
Subscales	Emotional pain, relational loss, absolute loss, emotional loss	Emotional support, instrumental support, religious, positive framing, acceptance	Only Coping Pattern III: Understanding the Health Situation used	Only Parental Control subscale used	Helplessness (not used in Chen et al., 2025), acceptance, perceived benefits
**Psychometric properties***					
Number of items	11	20; Brief 10	8	2	18
Cronbach's alpha	0.85<br>Subscales: 0.55-0.85	0.77	0.70	N/A – only two items, r = 0.43	Subscales: 0.856 – 0.875
Test-retest reliability	Not reported	Not reported	Not reported	Not reported	Not reported
Demonstrated sensitivity to change?	Yes	Not reported	Not reported	Not reported	Not reported
Demonstrated validity?	Not reported	Not reported	Not reported	Not reported	Not reported
Conducted factor analysis?	Not reported	Yes	Not reported	Not reported	Not reported

**Measure name**	***Illness Perception Questionnaire Revised for ASD***	**Impact of Event Scale - Revised**	***Locus of Control Scale***	***Parental Acceptance and Understanding of Autistic Children***	**Parents' Perception of Uncertainty in Illness Scale**
Original citation	*Mire et al., 2018*	Creamer et al., 2003	Cappé et al, 2011	Lee et al., 2024	Mishel & Epstein, 1990
Adapted from	Moss-Morris et al., 2002; Weinman et al., 1996	N/A	Cousson-Gélie et al., 2005	N/A	N/A
Construct measured	Perceptions about autism	Post-traumatic stress symptoms associated with autism diagnosis	Locus of control regarding autism	Acceptance of autism	Uncertainty regarding autism
Number of studies used	7 (e.g., Grebe et al., 2022; Rosenbrock et al., 2021)	1 (Nguyen et al., 2016)	3 (e.g., Cappé et al, 2018; Cappé et al., 2024)	2 (Lee et al., 2024; Lee et al., 2025)	1 (O'Brien, 2016)
Languages available	English, Persian (Norozi et al., 2023)	English	French	English	English
Type of measure	Questionnaire	Questionnaire	Questionnaire	Questionnaire	Questionnaire
Subscales	Timeline (acute/chronic), timeline cyclical, consequences, personal control, treatment control, illness coherence, emotional representation	Intrusion, avoidance, hyperarousal	Perceived control over the onset; perceived control over development; irrational beliefs regarding the disorder	Understanding, innate, acceptance, expectations	Ambiguity, lack of clarity, lack of information, unpredictability
**Psychometric properties***					
Number of items	38	22	17	30	31
Cronbach's alpha	Subscales: 0.40-0.91	0.94	Subscales: 0.60-0.77	Subscales: 0.47-0.86	0.86
Test-retest reliability	Subscales r = 0.194-0.884	Not reported	Not reported	Yes	Not reported
Demonstrated sensitivity to change?	Not reported	Yes	Not reported	Yes (innate subscale only)	Not reported
Demonstrated validity?	Not reported	Not reported	Not reported	Yes	Not reported
Conducted factor analysis?	Yes	Not reported	Yes	Yes	Not reported

**Measure name**	**Positive Gain Scale**	**Post-Traumatic Growth Scale**	**Questionnaire on Resources and Stress – Pessimism Subscale**	**Reaction to Diagnosis Interview**
Original citation	Jess et al., 2020	Tedeschi & Calhoun, 2004	Friedrich et al., 1983	Pianta & Marvin, 1993
Adapted from	N/A	Tedeschi & Calhoun, 1996	N/A	N/A
Construct measured	Positive contribution of autism to life	Posttraumatic growth/positive contribution of autism to life	Pessimism about the child	Resolution of autism diagnosis
Number of studies used	1 (Lunsky et al., 2021)	7 (e.g., Ebrahim & Alothman, 2021; Feng et al., 2022)	1 (Abbedutto et al., 2004)	13 (e.g., Dolev et al., 2016; Hutman et al., 2009)
Languages available	English	English, Arabic (Ebrahim & Alothman, 2021), Chinese (Wang et al., 2011), Spanish (Castro et al., 2015), Turkish (Cetinbakis et al., 2020)	English	English, Arabic (Dolev et al., 2016), Dutch (Poslawsky et al., 2014), Hebrew (Oppenheim et al., 20009), Italian (Leccisco et al., 2013), Persian (Norozi et al., 2023)
Type of measure	Questionnaire	Questionnaire	Questionnaire	Semi-structured interview
Subscales	None	Appreciation of life, personal strength, new possibilities, relating to others, self-transformation	Only Pessimism subscale used	None<br>Wachtel & Carter, 2008 – created coding scheme allowing for continuous measurement in addition to categorical classification
**Psychometric properties***				
Number of items	7	20/21 (different versions)<br>10 (Short-form; Castro et al., 2015)	11	5
Cronbach's alpha	0.77	0.91-0.96<br>Subscales: 0.63-0.95	0.72	N/A
Test-retest reliability	Not reported	Not reported	Not reported	Not reported
Demonstrated sensitivity to change?	No	No	Not reported	Yes
Demonstrated validity?	Not reported	Not reported	Not reported	Not reported
Conducted factor analysis?	Not reported	Not reported	Not reported	Not reported

**Measure name**	**Reaction to Diagnosis Questionnaire**	**Self-Blame and Responsibility Scale**	**Self-stigmatizing Thinking's Automaticity and Repetition Scale**	***Sense Making***	***Sense Making Scale for Parents of Children with Asperger's Syndrome***
Original citation	Sher-Censor et al., 2020	Mak & Kwok, 2008	Chan & Mak, 2017	*Pakenham et al., 2004*	*Samios et al., 2008*
Adapted from	Pianta & Marvin, 1993	N/A	N/A	N/A	N/A
Construct measured	Resolution of autism diagnosis	Self-blame for child's autism	Stigma processes	Making sense of autism	Making sense of autism
Number of studies used	4 (e.g., Leadbitter et al., 2025; Naicker et al., 2024)	1 (Lodder et al., 2020)	1 (Chan & Lam, 2018)	1 (Pakenham et al., 2004)	2 (Samios et al., 2012)
Languages available	English, Italian (Lecciso et al., 2025)	Chinese, English (Lodder et al., 2020)	Chinese	English	English
Type of measure	Questionnaire	Questionnaire	Questionnaire	Questionnaire	Questionnaire
Subscales	None	None	Repetition, automaticity	None	Spiritual sense making, causal attributions, changed perspective, identification, reframing, luck/fate
**Psychometric properties***					
Number of items	40 (Naicker et al., 2024);<br>42 (Levante et al., 2025; Sher Censor et al, 2020);<br>46 (Leadbitter et al., 2025)	Not reported	8	3	28
Cronbach's alpha	0.83-0.86<br>McDonald's $\omega = 1.20$	0.75	0.91<br>Subscales: 0.81-0.94	Not reported	0.68-0.89
Test-retest reliability	Not reported	Not reported	Not reported	Not reported	Subscales $r = 0.56 – 0.86$
Demonstrated sensitivity to change?	Yes	Yes	Not reported	No	Not reported
Demonstrated validity?	Not reported	Not reported	Yes	Not reported	Yes
Conducted factor analysis?	Yes	Not reported	Yes	Not reported	Yes

**Measure name**	**Societal Attitudes Towards Autism Scale**	**Stress-Related Growth Scale**	**Ways of Coping Checklist - Revised**	***Unnamed measure of changes to relationships and behaviour following diagnosis***	***Unnamed measure of parent reaction post diagnostic feedback session***
Original citation	Flood et al., 2013	Park et al., 1996	Cappé et al., 2011	*Al-Kandari et al., 2017*	*Anderberg & South, 2021*
Adapted from	N/A	N/A	Vitaliano et al., 1985; Cousson et al., 1996	N/A	N/A
Construct measured	Perceptions of autism	Positive contributions of autism to life	Coping strategies used following diagnosis	Changes to relationships and behaviour	Reaction to diagnosis
Number of studies used	1 (Colombo-Dougovito & Joshi, 2025)	1 (Alon et al., 2019)	1 (Cappé et al., 2018)	1 (Al-Kandari et al., 2017)	1 (Anderberg & South, 2021)
Languages available	English	English	French	Arabic	English
Type of measure	Questionnaire	Questionnaire	Questionnaire	Questionnaire	Questionnaire
Subscales	None	Personal growth, social growth, religious growth	Problem solving, emotional strategies, social support-seeking	None	Difficult emotions, preparedness, positive emotion, confusion
**Psychometric properties***					
Number of items	16	26	27	7	18
Cronbach's alpha	0.86	0.92<br>Subscales: 0.85-0.87	Subscales: 0.74-0.80	Not reported	Subscales: 0.75-0.86
Test-retest reliability	Not reported	Not reported	Not reported	Not reported	Not reported
Demonstrated sensitivity to change?	Not reported	Not reported	Not reported	Not reported	Not reported
Demonstrated validity?	Yes	Not reported	Not reported	Not reported	Not reported
Conducted factor analysis?	Not reported	Not reported	Not reported	Not reported	Yes

**Measure name**	***Unnamed measure of coping strategies used following diagnosis***	***Unnamed measure of response to diagnosis***	***Unnamed measure of caregiver acceptance of having an autistic child***	***Unnamed measure of family beliefs about autism***	***Unnamed measure of perception of autism diagnosis as a loss***
Original citation	*Ayyash et al., 2023*	*Colombo-Dougovito & Joshi, 2025*	*Kheir et al., 2012*	*Su et al., 2013*	*Wayment & Brookshire, 2018*
Adapted from	N/A	N/A	N/A	N/A	N/A
Construct measured	Coping strategies used following diagnosis	Reaction to diagnosis	Acceptance of autism	Perceptions of autism	Perception of autism as a loss
Number of studies used	1 (Ayyash et al., 2023)	1 (Colombo-Dougovito & Joshi, 2025)	1 (Kheir et al., 2012)	1 (Su et al., 2013)	1 (Wayment & Brookshire, 2018)
Languages available	English, Arabic	English	Arabic	Chinese	English
Type of measure	Questionnaire	Questionnaire	Questionnaire	Questionnaire	Questionnaire
Subscales	None	None	None	Not reported	None
**Psychometric properties***					
Number of items	5	1	2	Not reported	9
Cronbach's alpha	Not reported	N/A	Not reported	Not reported	0.84
Test-retest reliability	Not reported	Not reported	Not reported	Not reported	Not reported
Demonstrated sensitivity to change?	Not reported	Not reported	Not reported	Not reported	Not reported
Demonstrated validity?	Not reported	Not reported	Not reported	Not reported	Not reported
Conducted factor analysis?	Not reported	N/A	Not reported	Not reported	Not reported

**Measure name**	***Unnamed measure of perceived injustice of autism diagnosis***	***Unnamed measure of autism-related rumination***	***Unnamed measure of perceived cause of the child's autism***
Original citation	*Wayment & Brookshire, 2018*	*Wayment et al., 2019*	*Wynn et al., 2024*
Adapted from	Lepore et al., 1996	Several unspecified measures	N/A
Construct measured	Perception of autism as unfair	Autism-related rumination	Beliefs about the cause of autism
Number of studies used	1 (Wayment & Brookshire, 2018)	1 (Wayment et al., 2019)	1 (Wynn et al., 2024)
Languages available	English	English	English
Type of measure	Questionnaire	Questionnaire	Questionnaire
Subscales	None	None	None
**Psychometric properties***			
Number of items	2	4	14
Cronbach's alpha	N/A – only two items, r = 0.31	0.81	Not reported
Test-retest reliability	Not reported	Not reported	Not reported
Demonstrated sensitivity to change?	Not reported	Not reported	Yes
Demonstrated validity?	Not reported	Not reported	Not reported
Conducted factor analysis?	Not reported	Not reported	Not reported
