# Supplementary material for: Systematic review of measures and interventions for caregiver adjustment to child autism diagnosis
Source: Autism. 2026 Jan 10;30(4):866–83. doi: 10.1177/13623613251407305 (PMC13005899; doi:10.1177/13623613251407305)
Supplement: sj-pdf-4-aut-10.1177_13623613251407305 – Supplemental material for Systematic review of measures and interventions for caregiver adjustment to child autism diagnosis [file sj-pdf-4-aut-10.1177_13623613251407305.pdf]

Supplement Table 3. Interventions targeting caregiver adjustment to their child's autism diagnosis.

**Intervention name**	*Problem-Solving Skills Training*	*SOLACE*	*Sesame Street "See Amazing in All Children"*
Citation	Nguyen et al., 2016<br>Intervention citation: Nezu, 1986	Lodder et al., 2020	Anthony et al., 2020
Study design	Single group[b]	Randomised controlled trial	Single group[b]
Sample size, intervention	24	9	176
Sample size, control	N/A	8	N/A
Adjustment outcome measure	Impact of Event Scale – Revised (Creamer et al., 2003)	Affiliate Stigma Scale (Mak & Cheung, 2008);<br>Self-Blame and Responsibility Scale (Mak & Kwok, 2010)	Affiliate Stigma Scale (Mak & Cheung, 2008);<br>Unnamed measure of acceptance of Children with ASD (Harnum et al., 2007)
**Intervention features**			
Participants	Parents only	Parents only	Parents only
Mean child age (SD)	Not reported; range 2-5 years	5.91 years (2.59)	4.46 years (1.08)
Content	Problem-solving skills	Psychoeducation, cognitive restructuring and compassion focused techniques	Psychoeducation with an autism affirming focus
Format	Individual	Group	Self-directed
Setting	In-person or phone	In-person and online videoconference	Online
Length	8 sessions	8 sessions	1 week
**Intervention outcomes**			
Effectiveness: post-intervention	$p = 0.007$**<br>Effect sizes not reported	Affiliate stigma: $p = 0.004$**<br>Self-blame and responsibility: N.S[a]., $p > 0.05$<br>Effect sizes not reported	Affiliate stigma: N.S., partial eta squared = 0.011<br>Acceptance of children with ASD: partial eta squared = 0.025*
Effectiveness: follow-up	6-months post-baseline<br>$p = 0.004$**<br>Effect sizes not reported	6-week follow-up<br>Affiliate stigma: $p < 0.001$***<br>Self-blame and responsibility: N.S., $p > 0.05$<br>Effect sizes not reported	1-month post-baseline<br>Affiliate stigma: N.S., partial eta square = 0.004<br>Acceptance of children with ASD: N.S., partial eta squared = 0.004

[a]N.S. denotes non-significant finding.

[b]Problem-Solving Skills Training and "See Amazing in All Children" intervention studies both included comparison samples of caregivers of non-autistic children, which were not included in the current review. Effectiveness in caregivers of autistic children was measured in a single group.

**Intervention name**	*Mindfulness-Based Cognitive Therapy*	*Complicated Grief Intervention Program*	*Family-child reading picture book*
Citation	Lunsky et al., 2021	Bravo-Benítez et al., 2024<br>Intervention citation: Shear & Bloom, 2017	Yang et al., 2024
Study design	Single group	Randomised controlled trial	Single group
Sample size, intervention	21	14	139
Sample size, control	N/A	14	N/A
Adjustment outcome measure	Positive Gain Scale (Jess et al., 2020)	Caregiver Grief Scale (Meichsner et al., 2016); Post Traumatic Growth Inventory Short Form (Castro et al., 2015; Tedeschi & Calhoun, 1996)	Affiliate Stigma Scale (Mak & Cheung, 2008)
**Intervention features**			
Participants	Parents only	Parents only	Parents and children only
Mean child age (SD)	20.92 years (5.32)	Not reported; range 6-12 years	Not reported
Content	Mindfulness-based cognitive therapy	Processing grief associated with child's autism	Shared book reading of a story about autism
Format	Group	Group	Group; home implementation
Setting	Online	In-person	In-person
Length	6 sessions	10 sessions	1 session; 4 weeks of home implementation
**Intervention outcomes**			
Effectiveness: post-intervention	N.S., $p > 0.05$	Caregiver grief scale: Emotional pain $p = 0.040$* Cohen's $d = 0.74$; relational loss $p = 0.030$* Cohen's $d = 1.29$;<br>absolute loss N.S. $p = 0.130$ Cohen's $d = 1.85$;<br>acceptance of loss $p = 0.020$* Cohen's $d = 0.88$<br>Post traumatic growth inventory: N.S. $p = 0.050$ Cohen's $d = 0.40$	N.S., $p = 0.513$
Effectiveness: follow-up	3-month follow-up<br>N.S., $p > 0.05$	N/A – no follow-up	N/A – no follow-up

**Intervention name**	*Empower-Autism*	*AutInsight*
Citation	Leadbitter et al., 2025	Lee et al., 2025
Study design	Randomised controlled trial	Randomised controlled trial
Sample size, intervention	255	20
Sample size, control	123	21
Adjustment outcome measure	Reaction to Diagnosis Questionnaire (Sher-Censor et al., 2020)	Parental Acceptance and Understanding of Autistic Children Scale (Lee et al., 2024)
**Intervention features**		
Participants	Parents only	Parents only
Mean child age (SD)	8.9 years (3.5)	6.41 (2.47)
Content	Psychoeducation, Acceptance and Commitment Therapy	Psychoeducation, Acceptance and Commitment Therapy (ACT) (Hayes, 2004; Hayes et al., 2011), attachment theory
Format	Group	Group
Setting	Online	Online
Length	5 sessions (3-hour sessions; 15 hours total)	5 sessions (2-hour sessions; 10 hours total)
**Intervention outcomes**		
Effectiveness: post-intervention	N/A – adjustment assessed at follow-up timepoint only	N.S., $p > 0.05$.<br>Cohen's $d = 0.12$.
Effectiveness: follow-up	52 weeks post-baseline<br>$p = 0.016$*<br>Cohen's $d = -0.23$	10 weeks after post-treatment assessment<br>N.S., $p > 0.05$<br>Cohen's $d = 0.26$.
